# Stability and Efficacy of Mucoadhesive Eye Drops Containing Olopatadine HCl: Physicochemical, Functional, and Preclinical In Vivo Assessment

**DOI:** 10.3390/pharmaceutics17040517

**Published:** 2025-04-15

**Authors:** Anđelka Račić, Veljko Krstonošić, Ana Micov, Uroš Pecikoza, Vladimir Dobričić, Erna Turković, Danina Krajišnik

**Affiliations:** 1 Department of Pharmacy, Faculty of Medicine, University of Banja Luka, Save Mrkalja 14, 78000 Banja Luka, Bosnia and Herzegovina; 2 Department of Pharmacy, Faculty of Medicine, University of Novi Sad, Hajduk Veljkova 3, 21000 Novi Sad, Serbia; veljko.krstonosic@mf.uns.ac.rs; 3 Department of Pharmacology, University of Belgrade—Faculty of Pharmacy, Vojvode Stepe 450, 11221 Belgrade, Serbia; ana.micov@pharmacy.bg.ac.rs (A.M.); uros.pecikoza@pharmacy.bg.ac.rs (U.P.); 4 Department of Pharmaceutical Chemistry, University of Belgrade—Faculty of Pharmacy, Vojvode Stepe 450, 11221 Belgrade, Serbia; 5 Department of Pharmaceutical Technology and Cosmetology, University of Belgrade—Faculty of Pharmacy, Vojvode Stepe 450, 11221 Belgrade, Serbia; erna.turkovic@pharmacy.bg.ac.rs (E.T.); danina.krajisnik@pharmacy.bg.ac.rs (D.K.)

**Keywords:** olopatadine hydrochloride, hydroxypropyl guar gum, sodium hyaluronate, eye drops, rheological synergism, mucoadhesivity, stability, ocular itch test

## Abstract

**Background:** The incorporation of polymers into drug delivery vehicles has been shown to be an effective strategy to prolong the residence time of active ingredients in the precorneal tear film and to increase ocular bioavailability. **Objectives:** The aim of this study was to develop novel, viscous eye drops containing olopatadine (OCH) as the active ingredient, polysaccharides hydroxypropyl guar gum (HPG), and sodium hyaluronate (SH), individually, and in combination as functional polymers. **Methods:** Viscous eye drops containing 0.1% OCH in combination with HPG (0.25%) and SH (0.4%), were prepared and evaluated for their physicochemical properties, rheological behavior, mucoadhesion, and preliminary stability. A novel rheological method was used to evaluate the resistance of the eye drops under simulated blinking conditions. In vivo efficacy was evaluated using an ocular itch test in mice to compare the formulations with a commercial product. **Results:** The formulations remained stable and transparent, with physicochemical parameters within acceptable ranges. Rheological studies confirmed pseudoplastic flow, with the HPG-SH combination exhibiting enhanced viscosity and shear-thinning properties for prolonged retention in the eye. Mucoadhesion was highest in SH-HPG formulations. During simulated blinking cycles, eye drops containing a combination of SH and HPG polymers fully regained their initial viscosity during the resting periods. Preliminary stability studies indicate that the formulated eye drops exhibit satisfactory physicochemical stability under various storage conditions. In vivo, OCH-SH and OCH-HPG-SH drops provided prolonged antipruritic and analgesic effects compared to the reference product. **Conclusions:** Polysaccharide-based innovative formulations improve OCH retention, enhancing therapeutic efficacy and patient compliance in the treatment of allergic conjunctivitis.

## 1. Introduction

Allergic conjunctivitis is the most common form of allergic eye disease, with seasonal allergic conjunctivitis accounting for approximately 50% of allergic conjunctivitis cases. When the ocular surface of hypersensitive patients comes into contact with an external antigen, it produces a type I, Ig E-mediated hypersensitivity reaction. As a result of binding to allergens, IgE can activate certain immune cells, such as mast cells, and trigger a series of immune responses, which can cause itching, tearing, pain, and in severe cases, even loss of vision. Furthermore, ocular allergies often overlap with other eye disorders, such as infections and dry eye disease. According to guidelines and consensus documents, topical antihistamines, mast cell stabilizers, or dual-acting drugs should be the first-line treatment for the treatment of allergic conjunctivitis [1,2].

Olopatadine hydrochloride is classified as a dual-action drug because it acts as a selective histamine (H1) receptor antagonist with mast cell-stabilizing properties. It has also shown the ability to inhibit various cytokines [2]. Although it is considered a well-tolerated and effective drug for the treatment of allergic conjunctivitis, the low ocular availability of all topically applied drugs to the eye poses another challenge for researchers [2,3,4].

Topical application is the preferred method for treatment of ocular allergies, mainly due to its ease of application and non-invasive nature. However, this route is associated with challenges as various protective mechanisms, such as the eyelids and tear film, eliminate the medicine following application. Furthermore, blinking and lacrimation are physiological mechanisms of eye protection, which are mainly responsible for the limited contact time between the applied product and the cornea, conjunctiva, and sclera and the resulting poor ocular availability. Therefore, repeated applications are often necessary to achieve the desired therapeutic effect [2,5]. As a result, overcoming these challenges to ensure effective drug delivery has become a highly challenging area of research in ophthalmology. These efforts have focused on strategies to prolong the residence time of drugs on the ocular surface [5,6]. Although numerous formulations have been developed, eye drops remain the most commonly used formulation for the topical treatment of ocular diseases because they are easy to prepare, fast-acting, and accepted by patients. The main strategy to optimize eye drops as a dosage form is to develop simple viscous solutions that remain stable after administration without changing their composition. The addition of a viscosity enhancer helps to increase the viscosity, stabilize the drug, and reduce its elimination rate from the eye. These relatively low molecular-weight polymers remain in the tear film, maintaining enhanced viscosity due to their low diffusivity and limited permeation into the biological membranes of the eye [7].

Mucoadhesivity can be a valuable property of eye drops, as it prolongs the residence time of the drug on the cornea. The mucus layer on the cornea contains various functional groups that provide opportunities for hydrogen bonding with hydrophilic mucoadhesive polymers. The ideal molecular weight and concentration of the mucoadhesive depends on the specific polymer used for drug delivery [7,8]. The addition of mucoadhesive polymers such as hyaluronic acid can maintain and prolong drug retention and enhance the therapeutic effect.

Hyaluronic acid is a naturally occurring glycosaminoglycan that is widely distributed in connective, epithelial, and nerve tissue. Its viscoelastic, hydration, and wound-healing properties make it an ingredient in eye drops. At physiological pH, HA is anionic, affecting its chain conformation, solubility, and rheology [5,6].

Hydroxypropyl guar gum, a hydrophilic nonionic derivative of guar gum, is popular in ophthalmic formulations. Its controlled etherification with propylene oxide enhances its performance, with its degree of molar substitution and molecular weight governing its intermolecular interactions and overall functionality [9].

This study was conducted to formulate viscous eye drops containing polysaccharides, the viscosity enhancer hydroxypropyl guar gum, and the mucoadhesive polymer sodium hyaluronate, either individually, or in combination for the topical ocular application of olopatadine to improve therapeutic outcomes in ocular allergies. In addition, the study aimed to investigate the feasibility of innovative in vitro and in vivo approaches to evaluate the quality and efficacy of optimized formulations with antihistamines. It is expected that optimized olopatadine eye drops would suppress allergic-like sensations more effectively than the reference formulation in an ocular itch test in mice, a well-established model of acute allergic conjunctivitis.

## 2. Materials and Methods

### 2.1. Materials

Olopatadine hydrochloride (further denoted as OCH) was kindly gifted from Hemofarm A.D. (Vršac, Serbia). Hydroxypropyl guar gum (HPG) with a measured molecular weight of 110 ± 7.5 kDa and a molar substitution of 0.6 was obtained from Solvay Chemicals GmbH (Rheinberg, Germany), while benzalkonium chloride was sourced from Fagron (Bornem, Belgium). Sodium hyaluronate (SH), with a measured molecular weight of 185 kDa, was generously supplied by Sandream Impact, LLC (Fairfield, NJ, USA). Porcine mucin type II was provided by Sigma-Aldrich (St. Louis, MO, USA). Commercially available OCH eye drops (Opatanol^®^, 1 mg/mL) (Novartis Pharma Services AG, Basel, Switzerland) were used for comparison. The composition of the commercially available preparation is listed in Table S1 (Supplementary Materials). All other reagents and solvents were high-performance liquid chromatography (HPLC) or analytical grade. The purified water used for experiments was filtered through a 0.22 µm nylon membrane filter before use.

### 2.2. Animals

All procedures were conducted following the European Communities Council Directive 2010/63/EU on the protection of animals used for scientific purposes and were approved by the Institutional Animal Care and Use Committee of the Faculty of Pharmacy, University of Belgrade. Male C57BL/6 mice (weighing 20–30 g) used in this preclinical study were obtained from the Military Academy Breeding Facility, Belgrade, Serbia. Upon arrival, the animals underwent a one-week acclimatization period before the start of the experiments. Mice were housed under standard laboratory conditions, including a temperature of 22 ± 1 °C, 60% relative humidity, and a 12/12 h light/dark cycle. Food and water were provided ad libitum, except during the experimental procedures. To minimize diurnal variability in behavioral responses, all experiments were performed between 8 AM and 4 PM. A total of 108 mice were used in this study.

### 2.3. Preparation of OCH Polysaccharide Eye Drops

Eye drops were formulated containing OCH at a concentration of 0.1%, in combination with other excipients (Table 1). SH (in a concentration of 0.4% (w/V)), and HPG (in a concentration of 0.25% (w/V)) were incorporated to enhance viscosity and mucoadhesivity. Sodium chloride was used as a tonicity-adjusting agent, while dibasic sodium phosphate was used as a buffer component. Benzalkonium chloride (BAC) was used as a preservative, at the concentration recommended for ophthalmic formulations. In the first phase, a 1.0% (w/V) stock solution of OCH was prepared in purified water. In parallel, stock solutions of the viscosity-increasing agents were prepared by stirring the respective powders in purified water for 2 h at 300 rpm on a magnetic stirrer (2 mag magnetic motion MIX 15 eco, 2 mag-USA, Port Orange, FL, USA) to ensure uniform dispersion. The polymer solutions were then refrigerated for 24 h to allow complete hydration. In the second phase, solutions for the remaining excipients were prepared, which were then mixed with the previously prepared polymer dispersions. After mixing, the required amounts of prepared OCH solution and preservative were added. Before adding the required amount of purified water, the pH of the samples was carefully adjusted to a range of 5 to 6 by adding hydrochloric acid (0.1 mol/L) or sodium hydroxide solution (0.1 mol/L). Mixing continued until a homogeneous, viscous solution was obtained.

### 2.4. Physical Appearance, pH, and Osmolality Measurements

The appearance and clarity of the polymeric eye drops were examined for the presence of any particles or other particulate according to the Clarity and degree of opalescence of liquids test (Ph. Eur. 11.0; 2.2.1) [10]. Additionally, the optical transparency of the samples was determined spectrophotometrically using a two-beam spectrophotometer (UV-VIS, model 1800, Shimadzu, Kyoto, Japan). The transparency is the percentage ratio between the light that passes through a solution without being absorbed and the total light exposure. Absorbances (*A*) were measured at 480 nm, which correspond to the average wavelengths of human light perception [11]. The optical transparency of the tested samples (*T*) was calculated according to the following equation:

(1) $$A=2-\log_{10}\%T$$

The pH was measured using a digital pH meter (pH80+DHS, XS instruments, Capri, Italy) at 25 °C previously calibrated with standard buffer solutions of pH 4.0, 7.0, and 10.0. Measurements were performed in triplicate and the mean ± SD value were calculated for each formulation.

The osmolality of the OCH eye drops was determined using the single-sample freezing point osmometer Model 3320 Micro-Osmometer (Advanced Instruments, Inc., Norwood, MA, USA) equipped with a digital display and a freezing needle. A 20 µL sample was collected using the Ease-Eject™ Sampler, positioned in the instrument cradle, and inserted into the freezing chamber of the osmometer.

### 2.5. Surface Tension Measurement

The surface tension of the OCH eye drops was determined using a digital tensiometer (Easy DyneS K20/20023411, KRÜSS Scientific, Hamburg, Germany) following the du Noüy ring method at a constant temperature of 20 ± 0.1 °C. All measurements were automatically adjusted with the Harkins and Jordan correction factors incorporated into the KRÜSS tensiometer. Before measurement, the ring was immersed in the sample and left to equilibrate for 20 min. The reported values represent the average of five automatically repeated measurements.

### 2.6. Rheological Measurements

Rheological analyses of the viscous eye drops were carried out by HAAKE MARS rheometer (Thermo Scientific, Karlsruhe, Germany) equipped with a cone–plate C35 2°/Ti measuring system. Data collection and calculations were performed using the RheoWin 4.91.0011 software. Flow curves were obtained using a coaxial cylinder measurement system, with the shear rate ranging from 0.001 to 150 s^−1^ over 120 s (upward curve). The shear rate was then held at 150 s^−1^ for 60 s before gradually decreasing to 0.1 s^−1^ over the next 120 s (downward curve).

All curves were fitted using Ostwald-de Waele (power law) equation in order to determine flow parameters:

(2) $$\tau =K{\dot{\gamma }}^{n}$$

where τ(Pa) is the shear stress, $\dot{\gamma }(s^{-1})$ is the shear rate, *K* (Pa $s^{n})$ is a consistency index, and n (no dimensional) is the flow behavior index.

An additional rheological test was conducted to predict the behavior of formulated eye drops during eye blinking [12,13]. To simulate this process, the formulations were alternately exposed to a high shear rate of 4500 s^−1^ for 1 s (representing a single blink), followed by measurements at a low, non-destructive shear rate of 1 s^−1^ for 1 min. The high shear rate was selected to mimic the physiological shear rate of eye blinking, which ranges from 3000 and 40,000 s^−1^. The low shear rate was chosen to evaluate viscosity behavior during the inter-blinking period when the eye remains open. This cycle was repeated 10 times to simulate 10 blinking events. Although the natural inter-blinking period lasts between 5 and 7 s, a 1 min interval was used to ensure precise measurements.

During stability studies, viscosity was monitored using a rotational rheometer Rheolab MC 120 (Paar Physica, Stuttgart, Germany) equipped with the Z3 DIN concentric cylinder measuring device (25 mm). The shear rate was increased from 0 to 100 s^−1^ and then decreased from 100 to 0 s^−1^, with each step lasting 100 s. The apparent viscosity (mPa⋅s) was expressed as the mean of three measurements at 20 ± 0.1 °C [14].

### 2.7. In Vitro Mucoadhesive Strength

Mucoadhesiveness testing was carried out according to the previously described methodology with minor modifications. Mucoadhesive strength of the tested eye drops was measured using the EZ-LX texture analyzer (Shimadzu, Kyoto, Japan). Mucin disks were prepared by compressing 150 mg of porcine stomach mucin Type II using a ring press (11 mm in diameter) with a compression force of 10 tons applied for 30 s. Prior to testing, the mucin disks were hydrated by immersing them in simulated eye drops (Table S2, Supplementary Materials) for 20 s. Excess liquid was carefully removed using filter paper. The hydrated mucin disks were then securely affixed horizontally to the lower end of the insertion-jig acrylic probe (measuring 40 mm in length and 12.7 mm in diameter) using double-sided adhesive tape. The tested eye drops (at a temperature of 34 ± 1 °C) were placed in a glass beaker (with a diameter of 24 mm and a height of 35 mm), which contained 10 g of the sample to be tested. The measurement procedure was carried out by lowering the probe at a speed of 1 mm/s to the surface of the tested sample, with a pre-test force set to 0.005 N. After contact with the sample surface, at the same speed, the probe was immersed 2 mm into the tested sample and held in this position for 180 s to ensure adequate contact between the mucin disk and the sample. Subsequently, the probe was lifted out of the sample by vertical lifting for 10 mm at a speed of 0.5 mm/s. The TrapeziumX software (version 1.5.2) was used to record the force as a function of time. The maximum force required to detach the mucin disk from the sample represented the mucoadhesive force (mN), while the area under the force–time curve was estimated as the work of adhesion (mJ) [15,16]. The values of the above parameters were used to assess the mucoadhesive properties of the tested samples. All measurements were performed five times.

### 2.8. Stability Study of the Tested Eye Drops

The physicochemical stability of the formulated eye drops was tested at specific time intervals for a period of 12 months. The eye drops were stored in amber glass containers with high hydrolytic resistance in a refrigerator (4 ± 1 °C) and at an ambient temperature of 22 ± 2 °C. The parameters monitored for stability evaluation were appearance, clarity (visual), pH, osmolality, and drug content.

In addition, an accelerated stability study was performed using the heating/cooling cycle test. This test consisted of storing the samples at 40 ± 2 °C in the thermostat and then at 4 ± 1 °C in the refrigerator, maintaining each temperature for at least 48 h. A total of six alternating storage cycles were performed. At the end of the study, the physicochemical parameters (appearance, clarity, pH, osmolality, viscosity, and OCH content) were examined and compared with the initial values. The viscosity measurements were carried out 24 h after the last heating/cooling cycle.

### 2.9. Drug Quantification

During stability studies, the concentration of OCH was monitored according to the previously described chromatography-tandem mass spectrometry (LC-MS/MS) method [4]. The analysis was performed on UHPLC (Ultra High-Performance Liquid Chromatograph) ACELLA (Thermo Fisher Scientific Inc., Madison, WI, USA), coupled with a triple-quadruple mass spectrometer TSQ Quantum Access MAX (Thermo Fisher Scientific Inc., Madison, WI, USA) with a heated electrospray ionization (HESI) interface. The column used for analysis was Zorbax Eclipse XDB C18 (150 mm × 4.6 mm, 5 μm par-ticle size). The mobile phase was composed of acetonitrile and 20 mM ammonium acetate (50:50, *v*/*v*) and its flow rate was 0.5 mL/min. The column temperature was set to 45 °C and the injection volume was 10 μL. OCH was detected and quantified in positive HESI mode (*m*/*z* = 338.2–247.1). Prior to injections, samples were diluted with the mobile phase and filtered through the nylon filter (0.45 µm pore size).

### 2.10. In Vivo Efficacy Study

The efficacy of advanced OCH eye drops compared to the reference formulation was evaluated using an ocular itch test in mice, a well-established model of acute allergic conjunctivitis [17,18]. Histamine dihydrochloride (Sigma-Aldrich Chem. Co.), a known pruritogen, was dissolved ex tempore in phosphate-buffered saline (PBS, pH = 7.4).

The mice were placed in plexiglass chambers (30 × 25 × 25 cm) for approximately 15 min before the test to acclimate to the experimental environment. After the habituation period, manually restrained mice received a unilateral instillation of a single drop of histamine solution (46 μg in 2.5 μL PBS) directly into the lower conjunctival sac using a micropipette. Following administration, each animal was placed in an individual observation chamber, where histamine-induced behaviors were recorded for the next 30 min.

The characteristic response to histamine exposure in mice includes scratching episodes, which indicate an itch sensation, while wiping behavior is associated with a pain sensation [4,14]. A scratching bout was defined as the mouse raising its ipsilateral hind paw, engaging in forceful, repetitive scratching of the ocular area, and ending with either placing the paw back on the floor or bringing it to its mouth. Due to the rapid occurrence of scratching movements, they were counted in terms of bouts. Similarly, a wiping bout was characterized by the mouse raising its ipsilateral forelimb, making a single sweeping motion across the ocular region, and then returning to its initial position. Behaviors such as unilateral wiping or scratching of the contralateral eye area, as well as simultaneous wiping with both forelimbs (grooming), were not included in the analysis. Only scratching and wiping directed toward the treated eye were quantified [4,14]. The investigators visually monitored the itch- and pain-related behaviors rather than relying solely on video recordings to minimize potential result misinterpretations. The study also examined the impact of polysaccharide-based carriers on the duration and intensity of olopatadine’s antipruritic and antinociceptive effects. The reference and optimized novel formulations were administered at three time points, 15, 60, and 120 min prior to histamine exposure, after which the animals were subjected to the same experimental protocol.

The numbers of scratching/wiping bouts were converted to the percentage of antipruritic (%AP) and antinociceptive (%AN) activity, according to the following formula:

(3) $$\%\mathrm{AP}\;\mathrm{or}\;\%\mathrm{AN}=\frac{control\;group\;average\;number\;of\;bouts-number\;of\;bouts\;of\;each\;mouse\;in\;the\;test\;group}{control\;group\;average\;number\;of\;bouts}\times 100$$

### 2.11. Statistical Analysis

Statistical analysis was conducted using SigmaPlot 11 (Systat Software Inc., Richmond, CA, USA). All experiments were conducted at least in triplicate, and results are presented as mean values ± standard deviation (SD). In vitro data were analyzed statistically using one-way analysis of variance (ANOVA) followed by a post hoc test (Tukey’s), with statistical minimal level of significance set at a *p* value of <0.05. Additionally, the results from the in vivo study are expressed as the mean number of scratching/wiping bouts ± SEM for groups of 6–11 animals. The differences between corresponding means were verified using a two-way analysis of variance (ANOVA) followed by Tukey’s HSD post hoc test, with a significance level set at *p* < 0.05.

## 3. Results and Discussion

### 3.1. Physical Appearance, pH, and Osmolality Measurements

The tested samples remained clear and homogeneous in appearance 24 h after preparation, without turbidity, suspended particles or impurities. Clarity is a fundamental quality parameter for ophthalmic preparations and becomes particularly critical when viscosity-increasing agents are added to the formulation. The test results shown in Table 2 indicate that the optical transparency of the tested samples was between 96.7 and 100%. Therefore, all formulations can be considered completely transparent (transparency > 90%). The results of the pH and osmolality tests are shown in Table 2. The pH value is one of the most critical parameters regarding quality of ophthalmic preparations and must be controlled. In ophthalmic formulations, the pH value has an influence on the stability and solubility of the active ingredient as well as on the general tolerability of the preparation on the eye. The pH value of the tested eye drops varied from 5.2 to 5.6 and, thus, was acceptable for ophthalmic applications [19]. There is no significant difference between HPG and SH in the pH value, while other formulations differ significantly (*p* < 0.05). The osmolality of all samples was significantly different (*p* < 0.05) but remained within the tolerated range for ophthalmic preparations. Solutions with an osmotic pressure equivalent to a 0.6–2.0% sodium chloride solution are considered well tolerated.

### 3.2. Surface Tension

The measured surface tension values (σ) of the investigated formulations of eye drops with OCH are shown in Table 2. Most excipients such as preservatives, solubilizers, and viscosity-adjusting agents, but also some drugs such as antazoline and tetracaine, affect the surface tension, mainly leading to its lowering [19,20]. The surface tension of eye drops is rarely considered important, although it can be a useful technique for assessing the compatibility of eye drops and tear fluid [20]. Data in the literature indicate that OCH has surface activity. The measured maximum surface tension for OCH (5 mM OCH concentration in buffer solution) was 38 mN/m [21]. The surface tension of the SH formulation does not deviate significantly from this value. Furthermore, statistical analysis revealed that the surface tension of the SH sample was significantly higher than that of the HPG and HPG-SH samples (*p* < 0.05), while no significant difference was observed between HPG and HPG-SH samples (*p* > 0.05). The surface activity of SH polymer has not been discussed in detail in the literature. In general, it is assumed that biological polyelectrolyte polymers such as SH have a weak surface activity. However, in a study by Rieberio et al. [22], the dependence of the surface tension of SH solution on the concentration was shown, and the surface tension of solution in the range of 0.05–0.35% (m/V), measured at 34 °C, decreased from ~65 mN/m to ~50 mN/m, indicating some surface activity. In contrast, formulations containing HPG, a polymer that is reported in the literature to have moderate surface activity [23] were less active. This is the reason why HPG-containing formulations have a slightly lower surface tension.

The surface tension of the tested eye drops is slightly lower than the physiological surface tension of tear fluid, making them particularly beneficial for treating dry eye syndrome. This condition is associated with elevated surface tension at the air/tear fluid interface, typically ranging from 44 to 53 mN/m [24]. In addition, eye drops with lower surface tension exhibit significantly improved wetting properties, blend more easily with tear film components, and spread effectively across the corneal surface. This enhances their retention time in the precorneal area, resulting in a prolonged and more pronounced therapeutic effect [11,25]. This approach is particularly important in the overlap between dry eye syndrome and allergic conjunctivitis [26,27].

### 3.3. Rheological Characterization

Ophthalmic preparations should either maintain the natural behavior of the tear film or influence it to the greatest extent possible. Due to the pseudoplastic properties of tear fluid, formulations that use pseudoplastic vehicles are preferable to those exhibiting Newtonian flow, in which viscosity remains constant regardless of shear rate variations.

**Figure 1:**
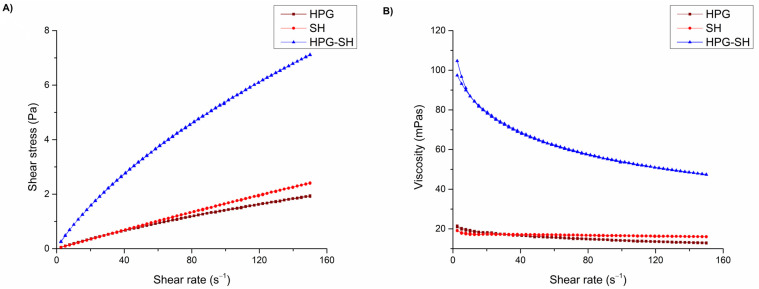
(**A**) Rheograms of the tested eye drops; (**B**) apparent viscosity as a function of shear rate.

Figure 1 shows the flow (A) and apparent viscosity (B) curves of the tested eye drops. It was determined that all tested eye drops show pseudoplastic flow. The results from Table 3 and Figure 1 confirm the shear-thinning character of the tested eye drops characterized by an initial high viscosity at very low shear rates, and followed by a viscosity decrease as the shear rate increased. The viscosity values obtained for the SH and SH-HPG eye drops were within the range recommended for ophthalmic preparations (15–50 mPa·s) and it is expected that these formulations are more resistant to the activities of the drainage system and show longer retention on the ocular surface, while being well tolerated by the eye [28]. In addition, the shear-thinning behavior provides significant advantages for ophthalmic formulations designed to remain on the ocular surface. It provides a high viscosity at rest when the eye is open, while allowing a reduction in viscosity under the mechanical shear exerted by blinking. This property minimizes irritation and discomfort and improves patient compliance and therapeutic efficacy [12]. Notably, the viscosity of SH-HPG eye drops is higher over a wide range of shear rates compared to formulations containing the polymers individually, as shown in Figure 1B. This observation confirms previous findings on the synergistic interactions between these polymers that contribute to the rheological properties of the formulation [14]. The rheological synergism between SH and HPG is probably due to their molecular interactions, which increase the viscosity of their aqueous solutions beyond what is observed for each individual polymer [29,30].

Additionally, Table 3 presents the consistency index (K) and the flow behavior (n) for the tested eye drops. The K coefficient reflects the system’s consistency and serves as an indicator of its viscosity. Meanwhile, the n constant defines the extent to which the system deviates from ideal Newtonian behavior. For a Newtonian fluid, n has a value of 1, whereas lower values indicate increasing shear-thinning non-Newtonian characteristics [31]. As can be seen, the index n was found to be less than 1 in all cases, confirming non-Newtonian, shear-thinning behavior. The HPG-SH formulation has a significantly (*p* < 0.05) lower n value, indicating a more pronounced shear-thinning behavior compared to HPG and SH. The consistency index (K) also reached its highest value for the HPG-SH formulation, indicating a greater apparent viscosity of the system. The differences in K between the formulations are statistically significant (*p* < 0.05). 

**Figure 2:**
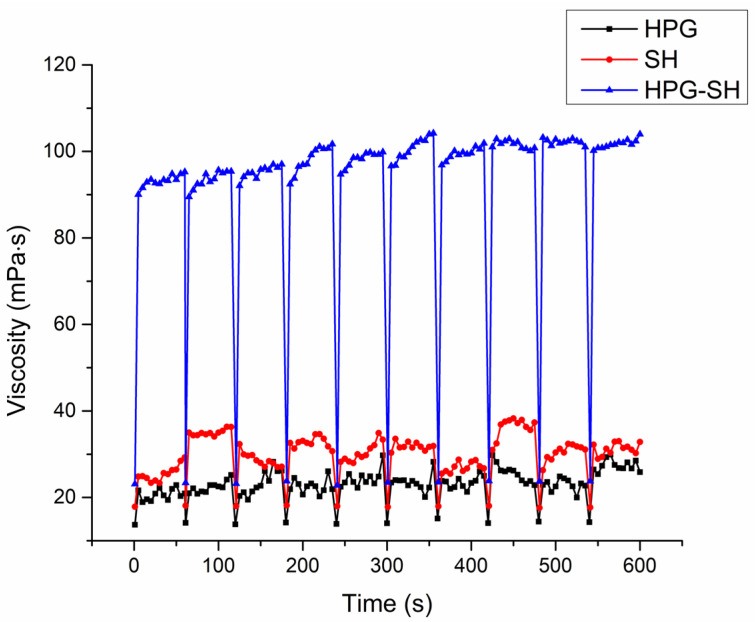
Viscosity values of the tested eye drops under simulated eye blinking.

In order to predict the rheological behavior of eye drops on the ocular surface, a further viscosity measurement was conducted at significantly higher shear rates. Based on eyelid velocity and tear film thickness (TF), the maximum shear rate exerted by the eyelid on the TF and the applied formulation varies between 0.03 and 4500 s^−1^ [12,32]. Furthermore, shear stress is expected to increase dramatically during blinking as the normal force increases due to eyelid movement [33]. However, the experimental determination of the viscosity of eye drops at such high shear rates poses a major challenge. Accurate measurement requires an extremely high angular velocity, which is difficult to achieve with conventional rheometric techniques [32]. In the current study, each blink was simulated at a shear rate of 4500 s^−1^ for 1 s and subjected to 10 consecutive simulated blinks. A 1 min rest period was maintained between consecutive blinks to allow sample recovery from the shear applied during the previous step and ensure accurate measurements. With each simulated blink, viscosities dropped to a minimum and then recovered during the inter-blink periods (Figure 2). High-viscosity fluids help decrease the drainage rate, thereby enhancing precorneal residence time. In summary, the pseudoplastic characteristics of these samples improved drug retention during the inter-blinking period, while shear thinning during the eye-blinking period prevented irritation [34].

After each application of the extremely high shear rate simulating blinking, the viscosity value dropped to approximately equal values of 13 mPa·s, 18 mPa·s, and 23 mPa·s for HPG, SH, and HPG-SH, respectively. Throughout all 10 simulated blinking cycles, eye drops containing a combination of SH and HPG polymers fully regained their initial viscosity during the resting periods. As shown in Figure 2, the SH-HPG formulation exhibits the highest viscosity, which is maintained even after repeated exposure to high shear rates. It can be expected that this eye drops formulation will be stable during blinking and successfully resist the drainage system, allowing prolonged contact with the corneal surface.

### 3.4. Mucoadhesive Strength of the Tested Eye Drops

Mucoadhesion strength tests play an important role in the evaluation of ophthalmic formulations, as higher mucoadhesive values can prolong the residence time of the drug on the ocular surface. The mucoadhesion strength of the tested eye drops was determined by measuring the force required to separate a mucin disk from the sample using a texture analyzer in tension mode. An applied force of 5 g facilitated direct and intimate contact between the partially hydrated mucin disk and the tested formulation, thereby initiating the initial contact phase of mucoadhesion. Based on a preliminary test, a contact time of 180 s was chosen, which provided sufficient time for adhesive bonds to consolidate through various interactions. The force required to detach the mucin disk from the formulation surface and the work of adhesion, were derived from the recorded force–time curve using the TrapeziumX software. The maximum values of mucoadhesive force and the work of adhesion values are presented in Table 4.

Among the tested formulations, the highest values for mucoadhesive strength (5.05 ± 0.21 mN) and work of adhesion (0.031 ± 0.006 mJ) were found for formulation, containing a combination of HPG and SH polymers. No significant difference was observed between formulations (*p* > 0.05). These results are consistent with previously published results on the mucoadhesive index, obtained using rheological methods when testing drug-free vehicles with the same polymer mixture [14]. It was previously determined that HPG does not exhibit pronounced mucoadhesive characteristics [35], which was confirmed by the mucoadhesive strength test. Therefore, the role of HPG in the formulation is to increase the viscosity and improve the viscoelastic properties of the formulation, enabling contact of the mucoadhesive polymer SH with the mucin on the surface of the eye. On the other hand, the results for formulation SH are consistent with previous findings, as the rheological method indicated pronounced mucoadhesive properties of the vehicle with the same polymer. This phenomenon can be explained by different theories of mucoadhesion. For separation measurements, the fracture theory, which assumes that adhesion failure only occurs at the interface between two surfaces, has proven to be the most suitable. However, this assumption is only valid if the mucin disk and the tested sample under study exhibit sufficient cohesiveness. It was concluded that mucoadhesive formulations should possess adequate cohesiveness to ensure a prolonged retention time [36]. The SH formulation had significantly lower viscosity values, which also had an effect on the cohesive properties. For a more accurate comparison of different polymers, studies with polymer formulations with similar rheological properties, such as corresponding viscosity profiles, should also be conducted [37].

### 3.5. Preliminary Stability of the Tested Eye Drops

The physicochemical characterization, which was carried out at defined time intervals within the storage period of one year under different conditions, aimed to evaluate the stability of the investigated formulations of eye drops with OCH. In addition, the stability of the tested formulations under stressful conditions was evaluated by performing heating/cooling cycles test. The values of the tested parameters under different storage conditions are shown in Table 5 and Table 6.

At the end of the storage period of the tested eye drop formulations under ambient conditions and at a temperature of 4 ± 1 °C (Table 5), no changes in appearance and clarity, such as cloudiness, precipitation of polymers, or of the drug, were observed. No significant changes were observed in the pH and osmolality of the tested eye drops stored at ambient conditions compared to the initial values.

The changes in pH values compared to the initial values were in the range of only ±3%, while the changes in osmolality were in the range of ±2%. The measured pH and osmolality values after one year of storage at a temperature of 4 ± 1 °C, also did not differ significantly from the initial values (Table 5). In addition, previous studies have shown that there are no significant interactions between the polymers used that could affect their physicochemical or functional properties, allowing their combination in the eye drops formulations [4,14]. The drug content was analyzed by HPLC using a validated method. After 12 months of storage under ambient conditions, the OCH content ranged between 90.00% and 98.11% (Table 5), which meets the requirements of the USP monograph Olopatadine Hydrochloride Ophthalmic solution (min. 90.0% and max. 110.0% of the declared amount of olopatadine) [38]. In the samples stored at a temperature of 4 ± 1 °C, the OCH content was in the range of 94.01–108.70% (Table 5), which also meets the requirements of the USP monograph. Based on the results obtained, it can be concluded that all tested formulations were stable under the specified test conditions.

After six cycles of temperature change from 4 ± 1 °C to 40 ± 2 °C with a residence time at each temperature of at least 48 h, the appearance, clarity, and color of all tested formulations remained unchanged (Table 6). Similarly to the previous storage conditions, the changes in pH were ±3% and osmolality ±2% with respect to the measured initial values. The measurement of viscosity before and after the cyclic temperature test showed insignificant changes in the apparent viscosity values, except for the formulation containing combinations of polymers. These findings are consistent with previous results [35], showing that the viscosity of HPG vehicles undergoes changes upon heating and cooling, i.e., a reversible change in viscosity occurs upon exposure of HPG to elevated temperatures [39]. The active ingredient content was also within an acceptable range of 93.46–109.9%.

The preliminary stability studies carried out indicate that the formulated eye drops have satisfactory physicochemical stability under all storage conditions, while the recommended storage conditions would be at a temperature of up to 25 °C.

### 3.6. In Vivo Efficacy Study

To test the influence of the polysaccharide-based carriers on the intensity and duration of OCH effects, we conducted an ocular itch test under histamine challenge, a model for acute allergic conjunctivitis. This test is considered a good paradigm for acute allergic conjunctivitis, given the shared features with the pathology of the anterior eye segment in humans. In addition, the ocular itch test is considered non-invasive, self-resolving, and an ethically favorable method, making it a valuable tool for preclinical investigation [4,14,17].

**Figure 3:**
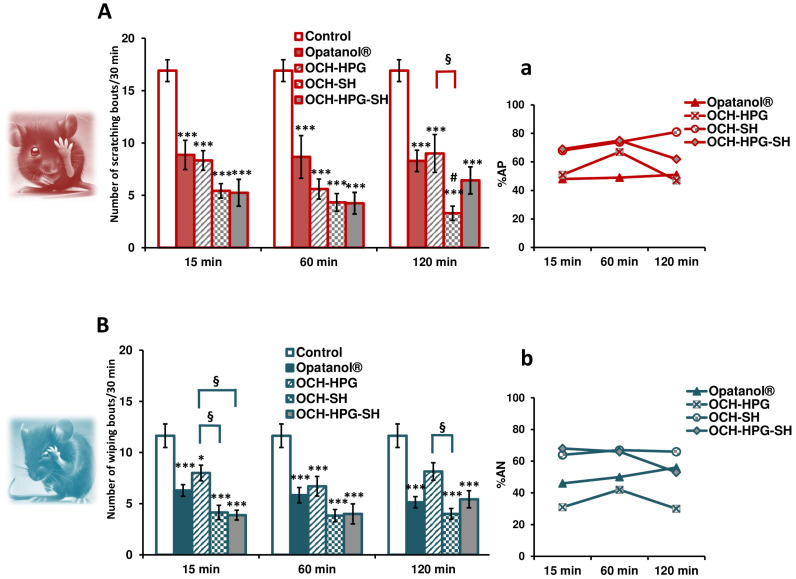
The effects of mucoadhesive eye drops with OCH (OCH-HPG, OCH-SH, and OCH-HPG-SH) and the reference product (Opatanol^®^) on pruritic-like and nociceptive-like behavior in the ocular itch test in mice. Results are expressed as the number of histamine-induced scratching (**A**) and wiping (**B**) bouts, along with the percentage of antipruritic (**a**) and antinociceptive (**b**) activity. The columns in panels (**A**,**B**) (from left to right) correspond to the order of treatment groups (from top to bottom) in the respective bar graph legends. Columns/points represent the group mean ± standard error of the mean (n = 7–12). Statistical analysis was performed using two-way analysis of variance (ANOVA) followed by Tukey’s post hoc test (* *p* < 0.05 and *** *p* < 0.001 compared to the control group; # *p* < 0.05 compared to the reference formulation; § *p* < 0.05 for comparison between OCH-SH or OCH-HPG-SH and OCH-HPG).

Following histamine instillation into the lower conjunctival sac, mice exhibited characteristic behavior indicative of itch and moderate pain (Figure 3A,B). In contrast, the histamine solvent (PBS) and polysaccharide-containing vehicles induced minimal responses. The primary endpoint—scratching behavior—served as an indicator of histamine-induced itch, resembling the histamine release from conjunctival immune cells in human allergic conjunctivitis. Since ocular pain can also occur, either as a symptom of allergic conjunctivitis or due to vigorous scratching (usually driven by the cornea, which is sensitive to painful stimuli), wiping behavior was assessed as a secondary endpoint, reflecting pain-related responses [18]. This pattern of response to histamine is consistent with histamine-induced itch and mild nociceptive sensation in humans [40].

Both the optimized and reference OCH eye drops significantly reduced histamine-induced scratching and wiping behaviors in mice, with all results reaching statistical significance (*p* < 0.001; two-way ANOVA). The key emphasis was placed on their antipruritic efficacy, with the mean values of scratching bouts recorded as follows: 8.9, 8.7, and 8.3 for Opatanol^®^ administered 15, 60, and 120 min before histamine instillation, respectively; 8.3 (15 min), 5.6 (60 min), and 9 (120 min) for OCH-HPG; 5.4 (15 min), 4.3 (60 min), and 3.3 (120 min) for OCH-SH; 5.3 (15 min), 4.3 (60 min), and 6.4 (120 min) for OCH-HPG-SH (Figure 3A). The corresponding antipruritic effects were 48–51% for Opatanol^®^, 47–67% for OCH-HPG, 68–81% for OCH-SH, and 62–75% for OCH-HPG-SH (Figure 3a). By increasing the time of drug administration before histamine, the antipruritic capacity of all eye drops remained relatively stable, with a tendency to increase over time with the OCH-SH formulation. The prolonged efficacy of the OCH-SH formulation suggests that SH alone may have a more sustained retention compared to the HPG-SH combination. While OCH-HPG-SH optimizes initial drug retention and spreading, OCH-SH alone may favor a more sustained drug delivery. Further studies could explore whether modifying the polysaccharide ratio in OCH-HPG-SH formulation could balance these effects for optimized long-term therapeutic benefits.

Both SH-based carriers (OCH-SH and OCH-HPG-SH) enhanced the antipruritic activity of OCH in comparison to the reference formulation, but the significance was reached only with OCH-SH eye drops when administered 120 min before the histamine challenge (Figure 3A). Notably, OCH-SH and OCH-HPG-SH formulations exhibited a rapid onset of action (both around 70%), maintaining high efficacy across all administration times (81% vs. 62%, respectively), which highlights their fast and sustained therapeutic effect, potentially allowing less frequent dosing and improving patient adherence.

These in vivo results align well with data obtained from the rheological and mucoadhesive analysis, confirming that OCH-SH and OCH-HPG-SH eye drops have enhanced viscosity, mucoadhesion, and ocular retention. As a result, these novel OCH eye drops may offer more effective and sustained relief from allergic symptoms.

Similar but less pronounced effects were observed for the antinociceptive activities of the tested OCH formulations. The corresponding analgesic-like effects were 46–56% for Opatanol^®^, 31–42% for OCH-HPG, 64–67% for OCH-SH, and 53–68% for OCH-HPG-SH eye drops. It could be assumed that SH-based OCH formulations are at least equally effective as the reference eye drops. Although statistical significance was not reached, the OCH-SH and OCH-HPG-SH eye drops might provide longer-lasting and/or more pronounced pain relief than commercially available products (Figure 3). This finding is clinically relevant, as reducing ocular pain could improve patient comfort in allergic conjunctivitis. Additionally, since eye pain is a known side effect of commercial OCH drops, these novel formulations may offer a dual benefit—enhancing efficacy while minimizing discomfort.

Overall, this preclinical study confirmed that polysaccharide-based vehicles significantly enhanced and prolonged both antipruritic and antinociceptive effects of OCH, likely due to rheological and mucoadhesive synergism, leading to prolonged ocular retention and sustained drug delivery.

### 3.7. Limitations and Future Directions

While this study provides valuable insights into the efficacy of polysaccharide-based OCH formulations in an acute allergic conjunctivitis model, several limitations should be acknowledged. First, although the histamine-induced ocular itch test is a well-established model of acute allergic conjunctivitis, it primarily reflects the early phase of an allergic reaction. This study did not assess the involvement of other inflammatory mediators, such as leukotrienes and prostaglandins, which contribute to later phases of allergic inflammation. Future studies should evaluate the efficacy of these formulations in chronic or repeated allergen exposure to better reflect clinical scenarios. Second, the dose–response relationship of the tested formulations was not assessed. Further experiments using multiple concentrations of the novel formulations are needed to determine the optimal dose for sustained efficacy. Third, long-term safety and tolerability remain to be investigated. While the formulations showed no signs of immediate irritation, chronic exposure studies evaluating ocular surface health, potential toxicity, and the risk of biofilm formation are important before translation to clinical use. Additionally, certain in vitro studies have inherent limitations. The method of assessing rheological behavior in the eye under laboratory conditions simplifies the actual physiological environment. For example, the study simulated a blink using a high shear rate of 4500 s^−1^ for 1 s, a value that, although within the lower end of the reported physiological range, may not fully capture the dynamic forces involved. Furthermore, the inter-blinking interval was modeled as a 1 min period at a low shear rate of 1 s^−1^, whereas the physiological inter-blinking period typically lasts only about 5–7 s. This extended low-shear phase could potentially lead to an overestimation of the time-dependent viscosity changes when compared to in vivo conditions. Future studies should include in vivo characterization to more accurately assess sustained release and retention time.

Despite these limitations, this preclinical study provides valuable proof-of-concept data that SH and HPG-SH-based carriers could enhance the therapeutic effects of OCH, supporting their potential for improved allergic conjunctivitis treatment with prolonged relief and better patient adherence.

## 4. Conclusions

The results of this study confirm that the tested ophthalmic formulations are stable and that their physicochemical properties are suitable for ocular application. All eye drop formulations exhibited high transparency, acceptable pH, and osmolality values, and remained stable under various storage conditions, including temperature fluctuations. The SH-HPG formulation exhibited pseudoplastic flow-behavior, the highest viscosity, and the strongest mucoadhesive properties, which overall contribute to longer residence time of the drug on the ocular surface and minimize irritation during blinking. In addition, in vivo studies using an ocular itch model demonstrated that polysaccharide-based formulations significantly improved the antipruritic and antinociceptive effects of olopatadine. The improved therapeutic efficacy observed in the SH and HPG-SH formulations compared to the reference product indicates that the mucoadhesiveness of the polymer, and not just the increased viscosity, is a more valuable property that contributes to prolonging the residence time of ophthalmic preparations.

## Figures and Tables

**Table 1:** Composition of the tested eye drops.

Composition % (w/V)	Formulation Code		
	HPG	SH	HPG-SH
OCH	0.1	0.1	0.1
HPG	0.25	-	0.25
SH	-	0.4	0.4
NaCl	0.6	0.7	0.7
Na_2_HPO_4_	0.5	0.5	0.5
BAC	0.01	0.01	0.01
Aqua purificata	q.s. ad 100 mL	q.s. ad 100 mL	q.s. ad 100 mL

**Table 2:** Eye drops’ physicochemical properties.

	Formulation Code		
	HPG	SH	HPG-SH
Appearance	Colorless	Colorless	Colorless
Transparency (%) *	96.7	100.0	98.4
pH *	5.5 ± 0.2 ^##^	5.6 ± 0.2	5.3 ± 0.1
Osmolality * (mOsmol/kg)	261 ± 1 ^##^	359 ± 2	378 ± 2
Viscosity (mPa·s) * at 150 s^−1^	12.89 ± 0.08	16.04 ± 0.10	47.37 ± 0.93
Surface tension ^#^ (mN/m)	34.3 ± 0.3	39.5 ± 0.1	34.2 ± 0.1

* Mean ± SD, n = 3; ^#^ Mean ± SD, n = 5; ^##^ from the published data [4].

**Table 3:** Apparent viscosity values, consistency index K, and flow behavior index n obtained by fitting the flow curves of the tested eye drops with Equation (2).

Viscosity * (mPa·s) at 20 °C	Formulation Code		
	HPG	SH	HPG-SH
25 s^−1^	17.56 ± 0.18	17.09 ± 0.02	75.05 ± 1.15
50 s^−1^	16.07 ± 0.06	17.05 ± 0.01	64.60 ± 1.30
100 s^−1^	14.13 ± 0.06	16.47 ± 0.14	53.53 ± 0.54
150 s^−1^	12.89 ± 0.08	16.04 ± 0.10	47.37 ± 0.93
K (Pa·s^n^)	0.0335 ± 0.0018	0.0205 ± 0.0001	0.1826 ± 0.0029
n	0.8114 ± 0.0122	0.9521 ± 0.0025	0.7331 ± 0.0007

* Mean ± SD, n = 3.

**Table 4:** Mucoadhesive strength and work of adhesion obtained for tested eye drops.

	Formulation Code		
	HPG	SH	HPG-SH
Mucoadhesive strength [mN] *	4.40 ± 0.30	4.37 ± 0.07	5.05 ± 0.21
Work of adhesion [mJ] *	0.021 ± 0.004	0.022 ± 0.004	0.031 ± 0.006

* Mean ± SD, n = 5.

**Table 5:** Values of the tested parameters for eye drops during 12 months of storage at ambient conditions (22 ± 2 °C) and at 4 ± 1 °C.

	Parameter	Storage at 22 ± 2 °C			Storage at 4 ± 1 °C		
		HPG	SH	HPG-SH	HPG	SH	HPG-SH
Initially	Appearance and clarity	Clear	Clear	Clear	Clear	Clear	Clear
	pH	5.2	5.6	5.3	5.2	5.6	5.3
	Osmolality (mOsm/kg)	261	359	378	261	359	378
	Drug content (%) *	106.15	101.13	111.06	109.03	104.52	114.11
3 months	Appearance and clarity	Clear	Clear	Clear	Clear	Clear	Clear
	pH	5.2	5.6	5.3	5.2	5.4	5.3
	Osmolality (mOsm/kg)	261	359	378	259	355	368
	Drug content (%) ^#^	106.15	101.13	111.06	99.08	94.23	103.51
6 months	Appearance and clarity	Clear	Clear	Clear	Clear	Clear	Clear
	pH	5.3	5.6	5.3	5.2	5.6	5.4
	Osmolality (mOsm/kg)	256	355	379	258	353	372
	Drug content (%) ^#^	90.57	106.93	89.19	95.41	90.38	94.74
12 months	Appearance and clarity	Clear	Clear	Clear	Clear	Clear	Clear
	pH	5.2	5.6	5.3	5.1	5.5	5.3
	Osmolality (mOsm/kg)	262	357	377	260	351	377
	Drug content (%) ^#^	94.62	98.11	90.00	98.90	98.46	107.63

* in relation to the declared content (the displayed values represent the mean value of three measurements; the coefficients of variation were less than 5%). ^#^ relative to the initial value (values shown are the mean of three measurements; coefficients of variation were less than 5%).

**Table 6:** Values of the tested parameters for eye drops before and after the heating/cooling cycles test.

	Parameter	Formulation Code		
		HPG	SH	HPG-SH
Before CT	Appearance and clarity	Clear	Clear	Clear
	pH	5.2	5.6	5.3
	Osmolality (mOsm/kg)	261	359	378
	Drug content (%) *	106.15	101.13	111.06
	Apparent viscosity (at 25 s^−1^)	77.6	77.2	94.2
After CT	Appearance and clarity	Clear	Clear	Clear
	pH	5.2	5.5	5.3
	Osmolality (mOsm/kg)	261	359	388
	Drug content (%) ^#^	108.49	109.90	100.00
	Apparent viscosity (at 25 s^−1^)	81.0	80.1	52.6

* in relation to the declared content (the displayed values represent the mean value of three measurements; the coefficients of variation were less than 5%). ^#^ relative to the initial value (values shown are the mean of three measurements; coefficients of variation were less than 5%).

## Data Availability

Data will be available on request.

## Supplementary Materials

The following supporting information can be downloaded at https://www.mdpi.com/article/10.3390/pharmaceutics17040517/s1, Table S1: Qualitative and quantitative composition of commercial Opatanol^®^ eye drops; Table S2: Qualitative and quantitative composition of the simulated tear fluid (STF).

## Abbreviations

The following abbreviations are used in this manuscript:

HPG	Hydroxypropyl guar gum
SH	Sodium hyaluronate
OCH	Olopatadine hydrochloride
BAC	Benzalkonium chloride
HPLC	High-performance liquid chromatography
%AP	Percentage of antipruritic activity
%AN	Percentage of antinociceptive activity
