# Supplementary material for: Stability and Efficacy of Mucoadhesive Eye Drops Containing Olopatadine HCl: Physicochemical, Functional, and Preclinical In Vivo Assessment
Source: Pharmaceutics. 2025 Apr 15;17(4):517. doi: 10.3390/pharmaceutics17040517 (PMC12030226; doi:10.3390/pharmaceutics17040517)
Supplement: Supplementary file 1 [file pharmaceutics-17-00517-s001.zip › pharmaceutics-3559142-supplementary.pdf]

Table S1. Qualitative and quantitative composition of commercial Opatanol® eye drops.

Olopatadine hydrochloride	1 mg/mL
Benzalkonium chloride	0.1 mg/mL
Sodium chloride	q.s.
Disodium phosphate dodecahydrate (E339)	q.s.
Hydrochloric acid (E507)	(to adjust pH)
Sodium hydroxide (E524)	(to adjust pH)
Purified water	q.s. ad 5 mL

Table S2. Qualitative and quantitative composition of the simulated tear fluid (STF).

Sodium chloride	6.7 mg/mL
Sodium bicarbonate	2 mg/mL
Calcium chloride dehydrate	0.08 mg/mL
Hydrochloric acid	(to adjust pH)
Purified water	q.s. ad 100 mL
